# Crosstalk between diurnal rhythm and water stress reveals an altered primary carbon flux into soluble sugars in drought-treated rice leaves

**DOI:** 10.1038/s41598-017-08473-1

**Published:** 2017-08-15

**Authors:** Seo-Woo Kim, Sang-Kyu Lee, Hee-Jeong Jeong, Gynheung An, Jong-Seong Jeon, Ki-Hong Jung

**Affiliations:** Graduate School of Biotechnology & Crop Biotech Institute, Kyung Hee University, Yongin, 17104 Korea

## Abstract

Plants retain rhythmic physiological responses when adapting to environmental challenges. However, possible integrations between drought conditions and those responses have not received much focus, especially regarding crop plants, and the relationship between abiotic stress and the diurnal cycle is generally not considered. Therefore, we conducted a genome-wide analysis to identify genes showing both diurnal regulation and water-deficiency response in rice (*Oryza sativa*). Among the 712 drought-responsive genes primary identified, 56.6% are diurnally expressed while 47.6% of the 761 that are down-regulated by drought are also diurnal. Using the β-glucuronidase reporter system and qRT-PCR analyses, we validated expression patterns of two candidate genes, thereby supporting the reliability of our transcriptome data. MapMan analysis indicated that diurnal genes up-regulated by drought are closely associated with the starch-sucrose pathway while those that are down-regulated are involved in photosynthesis. We then confirmed that starch-sucrose contents and chlorophyll fluorescence are altered in a diurnal manner under drought stress, suggesting these metabolic diurnal alterations as a novel indicator to evaluate the drought response in rice leaves. We constructed a functional gene network associated with the starch-sucrose KEGG metabolic pathway for further functional studies, and also developed a regulatory pathway model that includes OsbZIP23 transcription factor.

## Introduction

Plants, animals, and microorganism experience diurnal fluctuations in light and temperature that follow a 24-h cycle with diverse biological activities and physiological outputs^1–3^. This rhythmic pattern influences physiological behaviors including chronotype, diseases such as obesity and diabetes, the application of drug therapy, and cognitive functions in humans^2, 4^. Also, the diurnal rhythmicity of intestinal microbiota controls the transcription and epigenetic regulation of hosts^5^. In plants, it affects transcriptional regulation, hormone levels, stomatal conductance, photosynthetic carbon metabolism, nitrate and potassium uptake, metabolism, and overall growth and development^1, 6–11^. For example, bulk stomatal conductance is higher mid-day in the leaves of rice (*Oryza sativa*) and lower in the morning and evening due to variations in leaf chlorophyll concentration and solar radiation^9^. Research with *Zea mays* has demonstrated that the efficiency of diurnally varied uptake for nitrate, potassium, and phosphate differs among genotypes^10^. Promoter analyses have revealed that each phase in the regulatory module of ME/G-box, EE/GATA, and PBX/TBX/SBX controls transcriptional circuity^12, 13^. Rice is a major model system for which studies of functional genomics have made great advances. In fact, more than 50,000 genome-wide transcriptome data, 3000 whole-genome re-sequencing data, and 8646 QTLs have been obtained for those plants; its ease of transformation means that a gene-indexed mutant population is now available for almost half of this genome^14–21^. Other useful resources include datasets (GSE36040; GEO, http://www.ncbi.nlm.nih.gov/geo/) that cover diurnal rhythms under natural field conditions, with data collected from leaf blades at 2-h intervals over 2 d during each of nine developmental stages^22^.

Plants must also contend with abiotic stresses, such as water scarcities, that threaten global agriculture and food security due to a growing world population and climate change^23^. Crop productivity that solely depends upon rainfall is projected to decline as much as 50% by 2020, and yields in some countries might be reduced 90% by 2100^24^. Drought events now affect 20% of the land used for rice cultivation, i.e., 19 to 23 million ha (IRRI, http://www.irri.org). Immense economic losses related to drought mean that research of stress-tolerant crops is a major issue. The functions of 105 drought stress related genes have been characterized in rice, and are now part of the Overview of functionally characterized Genes in Rice Online (OGRO) database^25^. Although some investigations have focused on elucidating the mechanisms of drought tolerance, functions have been identified for only 3% of the related genes in the entire rice genome^26^.

Researchers have proposed that crosstalk exists in the relationship between abiotic stress and the diurnal cycle. In *Arabidopsis thaliana*, ABA signaling plays a role in regulating numerous genes that are drought-responsive in later daylight hours^27^. Other studies have also indicated a reciprocal relationship exists between the Salt Overly Sensitive (SOS) pathway and diurnal rhythms in rice, based on diurnal patterns of expression for *OsSOS1*, *OsSOS2*, *OsSOS3* and *RD29A* transcripts^28, 29^. In submerged rice plants, root extension and internal O_2_ concentrations in the roots exhibit relative fluctuations in their diurnal cycles when compared with plants that are not submerged. Ethanol production contributes to the survival of roots when those plants are submerged during the nighttime^30^. Moreover, approximately 75% more cold-responsive transcription factors (TFs) in *Arabidopsis* are induced in the morning than at night, and the diurnal cycle operates at a lower amplitude when temperatures are colder than normal^31^. That cycle also drives the highest cold-inducible Ca^2+^ signaling in guard cells and in whole plants in the middle of a photoperiod. The circadian clock is also engaged in whole-plant signaling^32^. Finally, rice *Cold induced MYB 1* (*CMYB1*) is thought to induce cold-responsive and rhythmic gene expression under nighttime temperatures^33^.

Despite those research advances, however, little has been done to improve our understanding of the relationship between diurnal rhythm and drought stress, and only two reports have been made about genome-wide analyses that examined the link between those components in *Arabidopsis* and in *Populus* ^27, 34^. Because abiotic stress is strongly affected by changes in the light/dark cycle and the temperature cycle, it is essential that diurnal rhythms should be included in such investigations of mutual interactions. Therefore, our objective here was to develop an effective means for studying how drought stress influences crosstalk with the diurnal cycle in rice. In particular, we examined key regulators of the drought-stress mechanism, and included “time of day” as one of our controlling factors. Global transcriptome analyses of genes involved in the drought response and in diurnal rhythms were combined. This allowed us to identify 766 genes that were then characterized through Gene Ontology enrichment and MapMan analyses. Combining our understanding of the predicted protein–protein interaction network with that of the starch-sucrose KEGG metabolic pathway provides a useful framework for further functional studies to elucidate the crosstalk between diurnal rhythm and the drought response. We obtained transcriptome data from drought-tolerant transgenic plants that over-express the OsbZIP23 transcription factor. By incorporating those data, we developed a regulatory pathway model that includes OsbZIP23 to explain this crosstalk.

## Results

### Genome-wide identification of rice genes showing both a drought response and diurnal regulation

**Figure 1:**
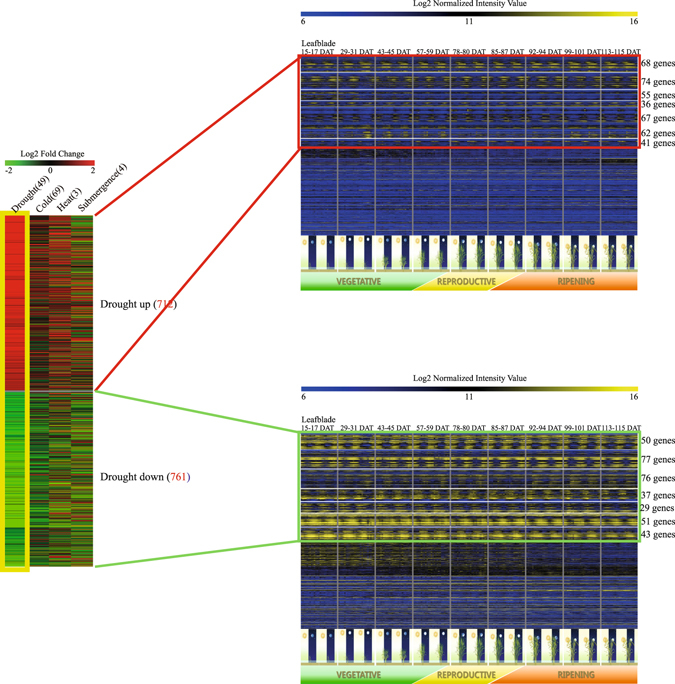
Identification and heatmap analysis of genes associated with both diurnal rhythm and drought response. Analysis (via KMC) of expression profiles for 766 genes with diurnal rhythm that are either up-regulated (403) or down-regulated (363) by drought. Average values for log 2 fold-changes under drought, cold, heat, or submergence conditions are shown in heatmap on left side. Drought-responsive genes are marked with yellow boxes. In two heatmaps on right side, log2-normalized intensity values indicate expression patterns for genes (red and green boxes) that were monitored at 2-h intervals (over 2-d period) using leaf blades from field-grown rice plants sampled during 9 developmental stages.

To determine which of our genome-wide candidate genes are drought-responsive, we used a meta-expression database for abiotic stress based on nine series of Affymetrix array data that comprised 131 samples (Fig. 1). Those data were downloaded from the National Center for Biotechnology Information (NCBI) Gene Expression Omnibus (GEO; http://www.ncbi.nlm.nih.gov/geo/) and were log2-normalized^35^. We then generated log2 fold-change data (expression in the stressed sample versus the MOCK control) for each replicate (Table S1). From this we identified 712 genes that were up-regulated under drought and 761 that were down-regulated, based on K-means clustering (KMC) in MultiExperiment Viewer (MeV) software^36^.

Diurnal patterns for these differentially expressed gene groups were further analyzed by using RXP_0002 data sets from the RiceXPro (RXP) website that were deposited as GSE36040 in the NCBI GEO. These data had been collected from the uppermost fully expanded leaf blades in the main stem of natural field grown rice plants at 2-h intervals for 2 d during nine developmental stages including the vegetative, reproductive, and ripening stages in 2008^22^. These data are helpful to observe diurnal rhythmic expression patterns according to the nature environment through entire growth stages. Analysis of KMC identified 403 genes showing diurnal rhythm plus upregulation under drought, while 363 genes showed diurnal rhythm with downregulation under drought (Tables S2 and S3). In detail, 34.99% of 403 genes exhibit the highest expression in the predawn; 29.53% of these show the midday peaks; 18.61% of these express the highest in the midnight; and 16.87% of these show the expression peak in the late day. The proportion of genes showing the expression peak in the predawn is also the highest for the latter 363 genes as 38.29%. The second largest percentage is 24.52% with regards to the midday time. The proportions of genes highly expressed during the midnight and the late day are 20.66% and 16.53%, respectively.

Among the remaining up-regulated genes, 35 were more highly expressed during the vegetative growth stage than during the reproductive growth stage, 16 were more highly expressed during the reproductive growth stage, and 258 had very low levels of expression or less-obvious diurnal patterns (Fig. 1). Among the remaining down-regulated genes, 110 showed strong expression during the vegetative growth stage but significantly reduced expression during the reproductive stage, 43 were more highly expressed during the reproductive growth stage than at any other stage, and 210 had less-clear expression patterns for diurnal rhythm. These data indicated that 56.6% (403/712) of the genes up-regulated by drought stress were also regulated by diurnal rhythm, while 47.7% (363/761) of the genes down-regulated by drought were also diurnally controlled. Thus, both groups of genes are a useful source for studying crosstalk between drought stress and diurnal regulation.

### Analysis of candidate genes with known functions using literature searches emphasizes their significance in promoting or maintaining crop yields when plants are subjected to abiotic stresses

To search for some of our candidate genes that have already been characterized through genetic and molecular studies, we utilized the OGRO database, which contains a good summary of rice genes with known functions^25^. There, we found that 53 of those candidates were functionally characterized previously (Table 1). Among them, 27 appeared to be involved in stress responses, including 11 that conferred drought tolerance. Four genes had roles in starch or sucrose biosynthesis, or in determining starch contents, while another eight had functions in grain yield. This demonstrated that genes showing both diurnal and water-stress responses might play key roles in promoting or maintaining crop yields in a world that is undergoing abiotic stresses.

**Table 1:** Information about functionally characterized genes from OGRO database, showing diurnal and drought-responsive patterns of expression.

Major Category	Drought Stress	Category of object character	MSU-ID	RAP-ID	Gene Symbol	Gene	Method of Isolation	Reference
R or T	Up	Drought and salinity tolerance.	LOC_Os02g50350	Os02g0736400	OsDHODH1	dihydroorotate dehydrogenase	Knockdown Overexpression	96
R or T	Up	Drought and salinity tolerance.	LOC_Os12g39400	Os12g0583700	ZFP252	zinc finger protein252	Overexpression	52
R or T	Up	Drought and salinity tolerance.	LOC_Os04g45810	Os04g0541700	Oshox22	—	Mutant	58
R or T	Up	Salinity tolerance.	LOC_Os07g47100	Os07g0666900	OsNHX1	Na+/H+ antiporter gene1	Overexpression	97
R or T	Up	Salinity tolerance, Ion homeostasis, Tolerance to oxidative stress.	LOC_Os01g64970	Os01g0869900	SAPK4	SNF1-type serine-threonine protein kinase4	Overexpression	98
R or T	Up	Abiotic stress response.	LOC_Os02g43330	Os02g0649300	OsSLI1	stress largely induced 1	Others	99
R or T	Up	Stomatal control, Resistance to oxidative stress.	LOC_Os03g12820	Os03g0230300	OsSRO1c	Similar to RCD One 1c	Mutant	100
R or T	Up	Resistance to *Magnaporthe grisea*.	LOC_Os11g03300	Os11g0126900	ONAC122	ONAC122	Knockdown	101
R or T	Up	Drought tolerance.	LOC_Os09g24990	Os09g0416800	OsCAF1G	rice carbon catabolite repressor 4(CCR4)-associated factor 1G	Others	102
R or T	Up	Tolerance to cold, drought, and salinity.	LOC_Os03g60560	Os03g0820300	ZFP182	zinc finger protein182	Overexpression	103
R or T	Up	Long-term acquired thermotolerance.	LOC_Os06g46900	Os06g0682900	HSA32	HEAT STRESS-ASSOCIATED 32-KD PROTEIN	Knockdown	104
R or T	Up	Drought and salinity tolerance, ABA sensitivity.	LOC_Os02g52780	Os02g0766700	OsbZIP23	basic leucine zipper23	Mutant	92
R or T	Up	Tolerance to drought and oxidative stress.	LOC_Os03g03370	Os03g0125100	dsm2	drought sensitive mutant2	Mutant	105
R or T	Up	Coleoptile elongation under submerged conditions.	LOC_Os11g10480	Os11g0210300	rad	reduced adh activity	Mutant	106
R or T	Up	Drought and salinity tolerance.	LOC_Os01g64730	Os01g0867300	Osabf1	ABA responsive element binding factor 1	Mutant	59
R or T	Up	Tolerance to drought and osmotic stress.	LOC_Os03g44150	Os03g0643300	OsOAT	Ornithine δ-aminotransferase	Overexpression	107
PT	Up	Lesion mimic, Leaf senescence.	LOC_Os10g25030	Os10g0389200	OsRCCR1	red chlorophyll catabolite reductase 1	Knockdown	108
PT	Up	Starch biosynthesis in endosperm, Amylopectin biosynthesis, Branch formation.	LOC_Os02g32660	Os02g0528200	SBE3	Branch enzyme isozyme	Others	53
PT	Up	Leaf senescence, Chlorophyll degradation.	LOC_Os09g36200	Os09g0532000	sgr	staygreen	Mutant	109
PT	Up	Grain length and width, 1000-grain weight, Flowering time.	LOC_Os01g15900	Os01g0264000	Rdd1	rice Dof daily fluctuations 1	Knockdown Overexpression	110
PT	Up	Pollen sterility, Homologous pairing.	LOC_Os12g04980	Os12g0143800	OsDMC1	-	Knockdown	111
PT	Up	Amylopectin content in seed.	LOC_Os06g06560	Os06g0160700	OsSSI	starch synthase I	Mutant	54
MT	Up	Dwarfism, Gibberellin catabolism.	LOC_Os07g01340	Os07g0103500	GA2ox5	Gibberellin 2-Oxidase5	Mutant	112
MT	Up	Pollen development, Anther dehiscence, Seed development.	LOC_Os04g39980	Os04g0475600	DAO	dioxygenase for auxin oxidation	Mutant	113
Others	Up	Starch content in culm.	LOC_Os05g50380	Os05g0580000	LSU3	ADP-glucose pyrophosphorylase large subunit 3	Mutant	114
Others	Up	Wounding response, salt-triggering ABA direct response, Seed-related legumains.	LOC_Os02g43010	Os02g0644000	OsaLeg2	Oryza sativa legumain 2	Others	115
R or T	Down	Cold and drought tolerance.	LOC_Os06g45140	Os06g0662200	OsbZIP52/RISBZ5	basic leucine zipper 52	Overexpression	116
R or T	Down	Resistance to *Magnaporthe grisea*.	LOC_Os03g18070	Os03g0290300	OsFAD7	fatty acid desaturase7	Knockdown	117
R or T	Down	Cold and drought tolerance.	LOC_Os02g33820	Os02g0543000	ASR3	ABA, osmotic stress, and ripening3	Overexpression	118
R or T	Down	Tolerance to phosphate starvation, Phosphate uptake.	LOC_Os03g05620	Os03g0150600	OsPT1	PHOSPHATE TRANSPORTER 1	Knockdown Overexpression	119
R or T	Down	Production of reactive oxygen species after treatment with flagellin in cultured cells.	LOC_Os08g35210	Os08g0453700	OsrbohE	respiratory burst oxidase homologsE	Knockdown	120
R or T	Down	Resistance to reactive oxygen species.	LOC_Os01g01660	Os01g0106400	OsIRL	isoflavone reductase-like	Overexpression	121
R or T	Down	Resistance to *Magnaporthe oryzae*, Cell wall defense.	LOC_Os06g03580	Os06g0125800	osbbi1	BLAST AND BTHINDUCED 1	Mutant	122
R or T	Down	Si translocation from root to shoot.	LOC_Os06g12310	Os06g0228200	lsi6	Low silicon rice 6	Mutant	123
R or T	Down	Tolerance to high manganese.	LOC_Os03g12530	Os03g0226400	OsMTP8.1	-	Mutant	124
PT	Down	Chlorophyll development.	LOC_Os03g20700	Os03g0323200	OschlH	-	Mutant	125
PT	Down	Photosynthetic capacity.	LOC_Os08g45190	Os08g0566600	PGR5	PROTON GRADIENT REGULATION 5	Knockdown	126
PT	Down	Promotion of flowering time under short days, Delay of flowering time under long days.	LOC_Os08g07740	Os08g0174500	Ghd8	grain number, plant height and heading date 8	Natural variation	127
PT	Down	Sterility, Cu transport.	LOC_Os04g45900	Os04g0542800	YSL16	Yellow stripe-like16	Mutant	128
PT	Down	Flowering time.	LOC_Os03g03070	Os03g0122600	osmads50	osmads50	Mutant	129
PT	Down	Biosynthesis of photosynthetic sucrose, Growth retardation.	LOC_Os01g64660	Os01g0866400	oscfbp1	cytosolic fructose-1,6-bisphosphatase1	Mutant	130
PT	Down	Metabolism of chlorophyll and carotenoids via methylation.	LOC_Os11g16550	Os11g0267000	OsGUN4	genomes uncoupled 4	Mutant	131
PT	Down	Carbon content.	LOC_Os07g04180	Os07g0134000	OsAAT60	amino acid transporter60	Mutant	132
PT	Down	Ammonium assimilation in leaves.	LOC_Os01g11054	Os01g0208700	Osppc4	Phosphoenolpyruvate carboxylase 4	Knockdown	133
PT	Down	Leaf senescence induced by reactive oxygen species.	LOC_Os10g37180	Os10g0516100	OsGDCH	H subunit of GDC	Knockdown	134
MT	Down	Internode elongation.	LOC_Os03g56950	Os03g0782500	OsPIL1	Phytochrome-interacting factor-like protein 1	Knockdown Overexpression	135
MT	Down	Panicle closure.	LOC_Os04g56170	Os04g0656500	OsLG1	liguleless 1	Natural variation	136
MT	Down	Dark-induced leaf senescence, Panicle color.	LOC_Os04g59610	Os04g0692600	SGRL	Stay-Green Rice like	Overexpression	137
MT	Down	Grain size.	LOC_Os05g06660	Os05g0158500	GS5	Grain size 5	Natural variation	138
MT	Down	Drooping leaf, Midrib development, Carpel identity.	LOC_Os03g11600	Os03g0215200	dl	DROOPING LEAF	Mutant	139
MT	Down	Dwarfism, Grain size, Leaf morphology.	LOC_Os03g13010	Os03g0232600	TUD1	Taihu Dwarf1	Mutant	140
Others	Down	Catalyzes key step in trigonelline biosynthesis.	LOC_Os02g57760	Os02g0823400	OMT	O-methyltransferase	Overexpression	141

R or T, resistance or tolerance; MT, morphological trait; PT, physiological trait; Up, candidate gene up-regulated under drought; Down, candidate gene down-regulated under drought; Method, type of mutant used in referenced study.

### Validation of expression patterns for drought-inducible and diurnally rhythmic genes, using the GUS reporter system

**Figure 2:**
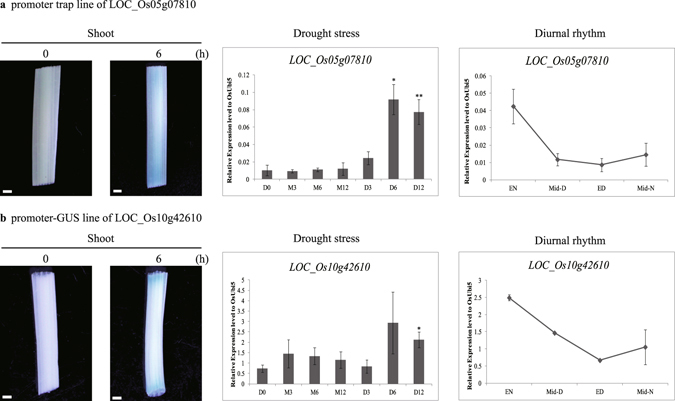
Validation of differential expression patterns under drought for two candidate genes using GUS reporter system and qRT-PCR. Results of qRT-PCR analyses: (**a**) drought-inducible GUS expression of *LOC-Os05g07810* by promoter trap system, and its expression level under drought stress and diurnal rhythm relative to *OsUbi5*; and (**b**) drought-inducible GUS expression of *LOC_Os10g42610* by promoter-GUS vector system, and its expression level under drought stress and diurnal rhythm relative to *OsUbi5*. D0, untreated leaf sample; M3, MOCK (Control) monitored for 3 h after drought stress; M6, MOCK after 6 h; M12, MOCK after 12 h; D3, leaf sample under drought for 3 h; D6, leaf sample under drought for 6 h; D12, leaf sample under drought for 12 h. For analyses of diurnal expression: EN, leaf sample collected at end of night; Mid-D, mid-day; ED, end of day; and Mid-N, middle of night. *OsUbi5* was used as internal control for qRT-PCR analysis. Error bars indicate standard errors of 3 replicates. *, **, ***, and ****: values are significantly different from each other at P ≤ 0.05, P ≤ 0.01, P ≤ 0.001, and P ≤ 0.0001, respectively.

To confirm the expression patterns of candidate genes up-regulated by drought, we used two strategies: screening of promoter trap lines (as we have described previously) and generation of transgenic plants carrying the β-glucuronidase (GUS) reporter gene vector under the control of the target gene promoter (Fig. 2)^37, 38^. For the former approach, we analyzed promoter trap lines for 70 candidate genes and found that only one had positive GUS expression that matched the activity of its co-segregating genotypes. PFG_3A-02918 is a promoter trap line of *LOC_Os05g07810*, which encodes a universal stress protein domain-containing protein. The T-DNA in this line is located in the first intron (Figure S1). Co-segregation tests of GUS activity and the T-DNA insertion confirmed that GUS expression was caused by the promoter trap of the target gene (Figure S1). In particular, GUS activity was observed in the shoots of 10-day-old seedlings that were subjected to drought stress for 6 h (Fig. 2a). Quantitative RT-PCR analysis revealed that *LOC*_*Os05g07810* expression was approximately nine times in the stressed plants than in the MOCK. Meta-analysis also showed that this gene followed a diurnal pattern, with expression being highest at the end of the night and lowest at the end of the day (Fig. 2a).

For the latter approach, we constructed promoter-GUS reporter gene vectors under the control of the *LOC_Os10g42160* promoter. Using T1 transgenic plants that expressed GUS, we tested inducible patterns under drought and found that, as we expected, GUS reporter activity was significantly stimulated by 6 h of stress (Fig. 2b). We also conducted quantitative RT-PCR analyses and confirmed that *LOC_Os10g42610* expression was approximately three times higher in stressed plants than in the MOCK control. Expression was again strongest at the end of the night and lowest at the end of the day (Fig. 2b).

The expression patterns of additional drought-inducible and diurnally rhythmic candidate genes encoding glycogen branching enzyme (OsSBE3, *LOC_Os02g32660*), Osβ-amylase3 (OsBAM3, *LOC_Os03g04770*), Osβ-amylase2 (OsBAM2, *LOC_Os10g32810*), Osβ-amylase5 (OsBAM5, *LOC_Os10g41550*), neutral/alkaline invertase (*LOC_Os04g33490*), and sucrose synthase (*LOC_Os03g22120*) were also validated (Figures S4 and S5). As expected, they all exhibited diurnal rhythmic pattern and drought induced expression. These expression data further enhance the fidelity of the global transcriptome data used in this study.

### Gene Ontology enrichment analysis reveals the significance of water-stress responses associated with diurnal regulation

**Figure 3:**
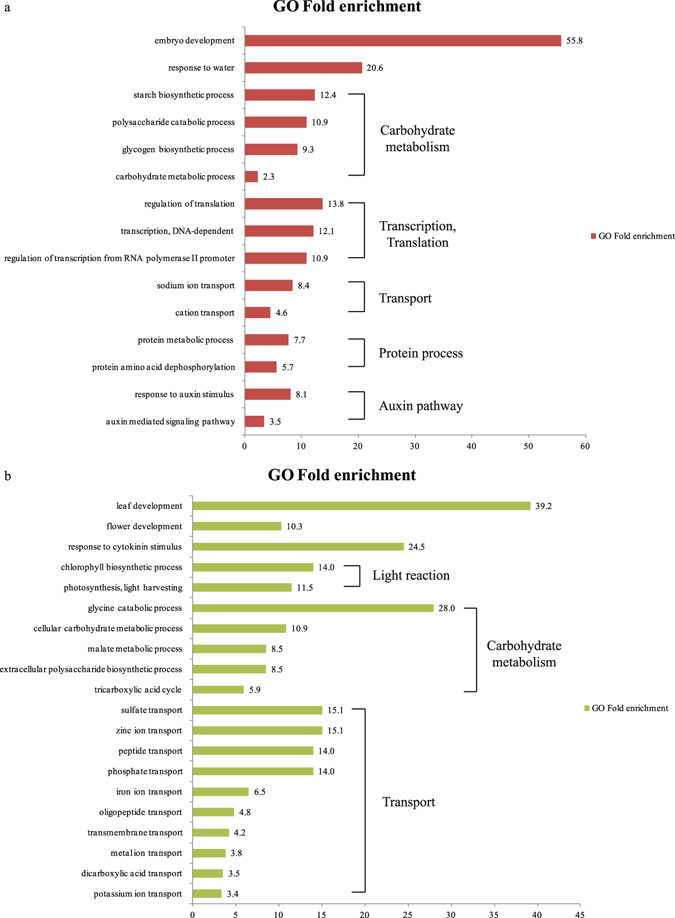
GO enrichment analysis of drought-responsive genes showing diurnal rhythm. Within category of Biological Process, GO enrichment terms are identified for 766 diurnally controlled genes that are either up-regulated (403; **a**) or down-regulated (363; **b**) by drought. Fold-enrichment values are shown along Y-axis; enriched GO terms, along X-axis.

We performed Gene Ontology (GO) enrichment analysis with the Rice Oligonucleotide Array Database (ROAD; www.ricearray.org/index.shtml) to identify the biological roles of candidate genes that are both drought-responsive and have a diurnal rhythm of expression. The GO terms were evaluated according to two thresholds (≥two-fold enrichment and P-value < 0.05)^39^. In all, 19 terms were significantly enriched for 403 genes. Among them, 15 were related to drought stress or diurnal rhythm (Fig. 3a, Table S4). Several of those terms could be organized into categories for carbohydrate metabolism, transcription and translation, transport, protein process, and auxin pathway. Highly overrepresented terms, such as those for embryo development (GO:0009790; by 55.8-fold), response to water (GO:0009415; by 20.6), starch biosynthetic process (GO:0019252; 12.4), polysaccharide catabolic process (GO:0000272; 10.9), glycogen biosynthetic process (GO:0005978; 9.3), and carbohydrate metabolic process (GO:0005975; 2.3) indicated that these biological processes were more likely associated with up-regulated drought responses and diurnal rhythms.

In total, 20 GO terms were enriched for 363 genes that were down-regulated by drought and also expressed diurnally. These terms covered categories for organ development, light reaction, carbohydrate metabolism, and transport (Fig. 3b, Table S5). Leaf development (GO:00048366; by 39.2-fold), flower development (GO:0009908; by 10.3), chlorophyll biosynthetic process (GO:0015995; 14.0), photosynthesis light harvesting (GO:0009765; 11.5), glycine catabolic process (GO:0006546; 28.0), cellular carbohydrate metabolic process (GO:0044262; 10.9), malate metabolic process (GO:0006108; 8.5), extracellular polysaccharide biosynthetic process (GO:0045226; 8.5), and tricarboxylic acid cycle (GO:0006099; 5.9) appeared to be important participants in the potential crosstalk between the diurnal cycle and drought-responsive downregulation.

### MapMan analysis reveals significant pathways associated with diurnal regulation and the drought response

**Figure 4:**
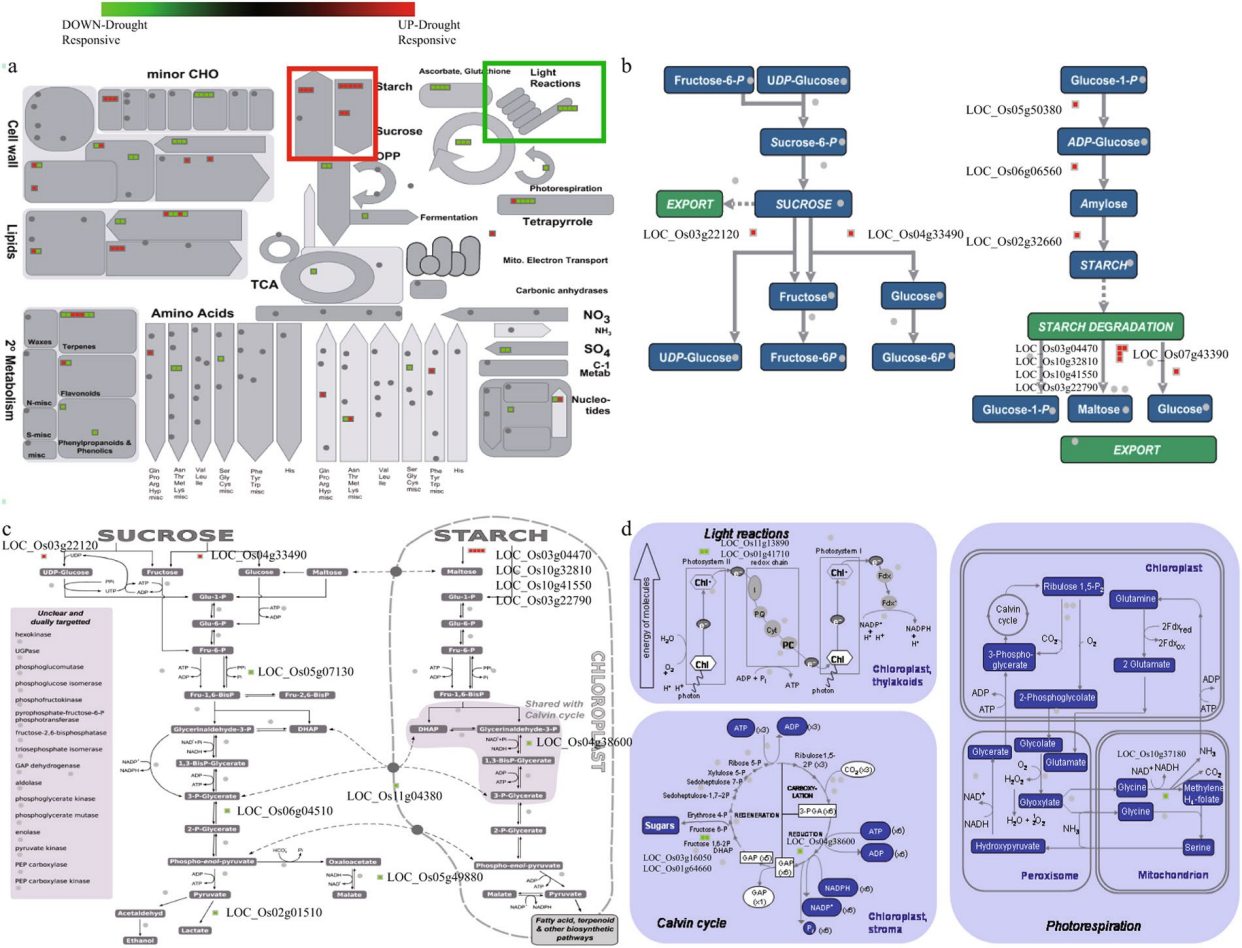
MapMan analysis of drought-responsive genes showing diurnal rhythm. Results of mapping 766 genes to Metabolism overview (**a**), Sucrose-Starch overview (**b**), Plant Glycolysis overview (**c**), and Photosynthesis overview (**d**). Red boxes, genes up-regulated under drought; green boxes, genes down-regulated under drought. Within Metabolism overview, red and green lines indicate MapMan functional groups selected for physiological validation.

To identify the metabolic pathways associated with drought and diurnal regulation, we conducted a MapMan Metabolism overview analysis for 766 candidate genes differentially expressed under drought stress as well as involved in diurnal rhythm. The Metabolism overview, installed in the MapMan toolkit (3.51R2), was executed as we have previously described (Fig. 4a, Table S6)^40^. From this we determined that pathways for starch biosynthesis and degradation, the sucrose degradation pathway, and lipid degradation metabolism were positively associated with drought and diurnal regulation. In contrast, light reactions and the Calvin Cycle in photosynthesis, pathways for ascorbate and glutathione, and the pathway for synthesis of cell wall precursors were negatively associated with this proposed crosstalk. We were also interested to find a strong positive association with the starch synthesis and degradation pathways. To get more detailed information about these pathways, we analyzed the Sucrose-Starch overview (Fig. 4b) and Plant Glycolysis overview (Fig. 4c) installed in the MapMan toolkit and identified detailed elements in the starch synthesis and degradation pathways that are associated with this crosstalk. Subsequently, we found up-regulated genes that encode ADP-glucose pyrophosphorylase (AGPase), starch synthase, and starch branching enzyme in the starch synthesis pathway, as well as upregulated genes for β-amlyases and disproportionation enzyme in the starch degradation pathway. Moreover, details about light reactions and the Calvin Cycle were identified in the Photosynthesis overview (Fig. 4d) and the Plant Glycolysis overview (Fig. 4c). We also discovered down-regulated genes, such as those for the light harvesting complex of Photosystem II (PSII) chlorophyll binding protein 1 (Lhcb1), chlororespiratory reduction 23 (CRR23), and proton gradient regulation 5 in photosystem light reactions, as well as glyceraldehyde 3-phosphate dehydrogenase A subunit (GAPA) and fructose-1,6-biophosphate in the Calvin Cycle. Based on these results, we speculated that the starch-sucrose pathway and photosynthesis are critical components of this crosstalk between drought stress and diurnal rhythm. Additional analyses covered overviews for regulation, transcription, large enzymes, cellular-responsive, secondary metabolism, receptor-like kinases, and proteasomes (Figures S7 and S8). Genes up-regulated by drought are marked in red while down-regulated genes are marked with green.

### Fluctuations in levels of soluble sugar and starch contents in rice leaves under drought stress during the day/night cycle

**Figure 5:**
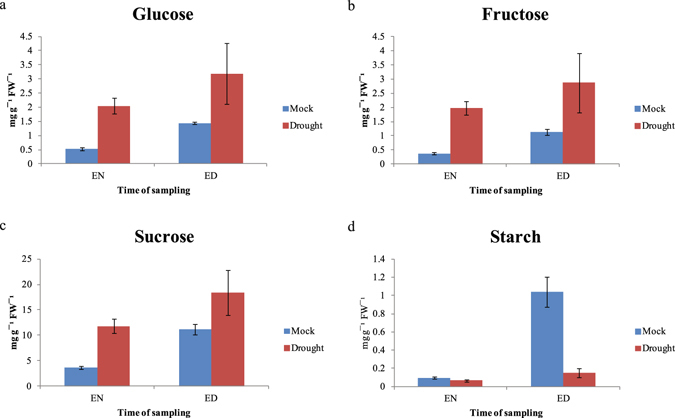
Diurnal alterations in soluble sugars and starch contents from rice leaves under drought stress. On Y axis: contents (mg g^−1^ FW of sample) of **a**) glucose; (**b**) fructose; (**c**) sucrose; and (**d**) starch. X-axis features time of sampling: EN, end of night; and ED, end of day. MOCK control, samples not subjected to stress treatment; Drought, samples from plants exposed to drought conditions. Error bars indicate standard errors of 3 replicates.

Rice plants were grown for five weeks in a controlled-climate chamber (28 °C/20 °C, 66% relative humidity, and 12-h photoperiod from incandescent lamps). Some of these plants were then exposed to drought stress for 9 d while the others remained under standard growing conditions (MOCK control). Leaves were sampled at the end of each dark period (night) and the end of each light period (day), and their soluble sugar and starch contents were measured by an enzymatic method, as described previously^41^. Under drought treatment, diurnal patterns of starch and soluble sugars, including sucrose, were altered, with soluble sugar contents tending to increase and starch levels decreasing in response to stress (Fig. 5)^42^. The soluble sugar contents peaked during the daytime^43^ and, in fact, were elevated even further as a result of drought treatment. By comparison, starch levels remained relatively low even at the end of the day, suggesting rapid turnover. These findings suggested that changes in sugar and starch contents could serve as a new indicator for evaluating the drought response associated with diurnal rhythm in rice leaves. When combined with the results from our genome-wide analysis presented above, the starch degradation pathway appears to have a particularly important role in altering the synthesis of those metabolites.

### Changes in chlorophyll fluorescence in rice leaves under drought stress during the day/night cycle

**Figure 6:**
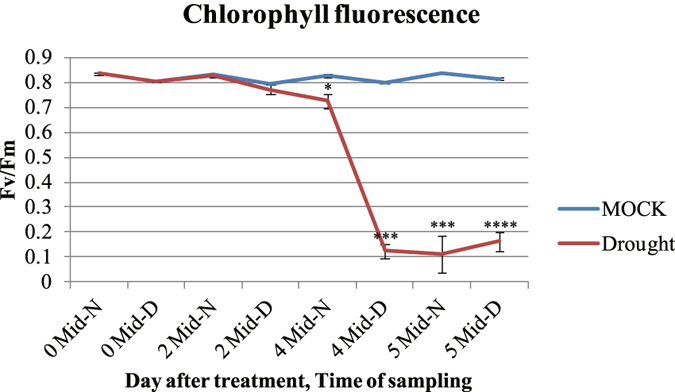
Diurnal alterations in chlorophyll fluorescence and total chlorophyll content in rice leaves under drought stress. Chlorophyll fluorescence (Fv/Fm) was measured in leaf samples from non-stressed MOCK plants (0) and drought-stressed plants (2, 4, and 5), collected mid-day (Mid-D) and mid-night (Mid-N) for periods indicated: 2 Mid-D and 2 Mid-N, sampled for 2 d; 4 Mid-D and Mid-N, sampled for 4 d; and 5 Mid-D and 5 Mid-N, sampled for 5 d. Error bars indicate standard errors of 3 replicates. *, **, ***, and ****: values are significantly different from each other at P ≤ 0.05, P ≤ 0.01, P ≤ 0.001, and P ≤ 0.0001, respectively.

Chlorophyll fluorescence (Fv/Fm)^44^ was measured mid-day and mid-night in 2-month-old rice plants that had been exposed to drought treatment for 0 to 5 d (Fig. 6). Although fluorescence is normally much lower mid-day than mid-night, the degree of difference is enlarged under a water deficiency^45–48^. We also found that mid-day values were drastically lower after 4 d of drought. When combined with the data gained in our analysis above, these findings led us to speculate that PSII in light reactions has a crucial role in this diurnal drought response, probably due to the water supply being limited.

### Construction of a functional interaction network focused on genes in the starch biosynthesis and degradation pathways for which expression shows both a drought response and diurnal rhythm

**Figure 7:**
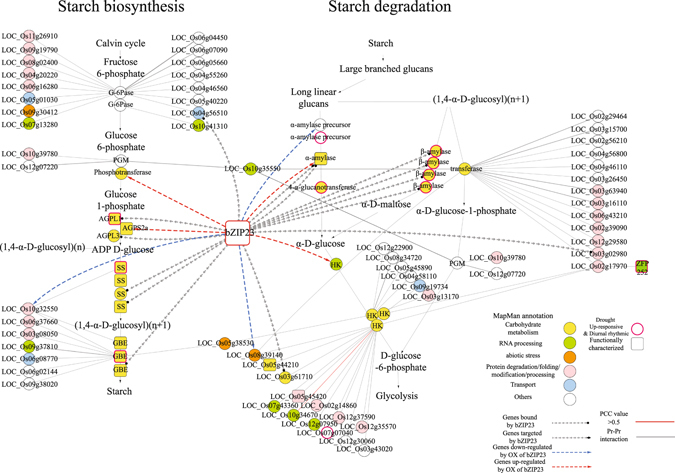
Analysis of functional gene network associated with pathways for starch biosynthesis and starch degradation. We used RiceCyc, Rice Interaction Viewer, and Cytoscape program to develop network, based on information derived from MapMan analysis. Nodes with bold-red borders, genes that are both diurnally rhythmic and up-regulated by drought; rounded rectangles, genes for which functions were reported previously. Colored nodes (circles) represent functional classification information from MapMan analysis (see Fig. 4), with color of edge indicating differential Pearson’s correlation coefficient (PCC) values that were calculated using anatomical microarray data from ROAD, and which ranged >0.5 (red). Thickness of edge was determined based on Interlog confidence value from Rice Interaction Viewer, with thicker edges indicating higher confidence. Dotted arrows with circle heads show genes encoding proteins bound by OsbZIP23; dotted lines with triangle heads, genes targeted by OsbZIP23. Blue-dotted lines, genes regulated negatively in overexpression of OsbZIP23; red-dotted lines, genes regulated positively in overexpression of OsbZIP23. Color indicates assigned function: yellow, carbohydrate metabolism; green, RNA processing; orange, abiotic-stress response; pink, protein degradation/folding/modification/processing; blue, transport; and white, other functions.

Because the starch biosynthesis and degradation pathways appear to be closely linked with the drought response and diurnal control, we explored the mechanism that might regulate this. To do so, we developed a functional interaction network mediated by 276 genes that encode the components of the entire starch biosynthesis and degradation pathways (Fig. 7, Table S7). These were derived from the RiceCyc Interaction Viewer (http://pathway.gramene.org/gramene/ricecyc.shtml) and the Rice Interaction Viewer (http://bar.utoronto.ca/interactions/cgi-bin/riceinteractionsviewer.cgi)^49, 50^. From this, we identified 69 interactions associated with six genes for starch biosynthesis and five for starch degradation. Within the network, 11 genes (nodes with bold-red borders in Fig. 7) were up-regulated by drought and also diurnally rhythmic. Six other genes had been functionally characterized previously^51–56^. Therefore, the interactions between them and genes not yet characterized might help us study the latter more closely. Through MapMan analysis of network components, we were able to assign functional roles for 15 genes associated with carbohydrate metabolism; eight for RNA processing, three for abiotic stress; four for transport metabolism; and 22 for protein degradation, folding, modification, or processing.

Among the genes assigned as TFs in the Transcription overview in MapMan analysis (Fig. 3b), three have known functions in drought tolerance: *Oryza sativa basic leucine zipper 23* (*OsbZIP23*, *LOC_Os02g52780*), *HD-Zip I homeobox 22* (*HOX22*, *LOC_Os04g45810*), and *ABA responsive element binding factor 1* (*ABF1*, *LOC_Os01g64730*)^57–59^. Because we could utilize only earlier results from CHIP-seq and RNA-seq analyses of OsbZIP23 TF, we integrated only genes for drought tolerance that are either regulated by or bound by the bZIP23 TF, according to this network^57^. In all, 11 genes appeared to be bound by OsbZIP23, with one being a direct target. Four genes were up-regulated and three were down-regulated by overexpression of *OsbZIP23* in the starch-sucrose metabolic pathway under drought conditions. Among them, five were diurnal and up-regulated by drought, similar to the pattern for *OsbZIP23*. These genes might be valuable for elucidating the roles of OsbZIP23 when linking the drought mechanism to starch-sucrose metabolism.

## Discussion

Plants challenged by drought must respond if they are to survive and continue to grow. Before deeper functional analyses can be conducted, researchers must first understand the possible connections between water stress and the diurnal cycle. This can be accomplished by using meta-expression data to identify differentially expressed genes^28^. Results from previous genome-wide transcriptome analysis have helped us discover the entire molecular network and examine stress-target genes and their complex responses in plants^60–62^. Here, we used microarray data to determine that 56.6% (403) of our 712 candidate drought-responsive genes are also diurnally expressed, while 47.7% (363) of the 761 genes down-regulated by drought are also diurnal. Hence, these 766 candidate genes provide fundamental materials for investigations of the reciprocal network between drought stress and diurnal rhythm. Because a large number of these drought-responsive genes are also diurnally rhythmic, we must consider what role the diurnal cycle has in that drought response. Probe sets for leaves from rice seedlings grown under normal conditions have indicated that only 10.96% of the rice transcriptome shows diurnal oscillations^6^. Because the percentage of rice genes that are both diurnally regulated and drought-responsive is higher than those that fulfill only one of those criteria, the extent to which drought stress influences these transcriptome rearrangements appears to be dependent upon the time of day. Similar results have been found with *Arabidopsis* ^27^. Regarding time phasing patterns, it is clearly seen that more than one third of drought responsive genes relatively show higher expression level during predawn. The phase distribution of diurnal rhythm might not be influenced by drought stress because the period from 2:00 am to 4:00 am has the highest proportion of probe sets showing diurnal rhythmic distribution in rice seedling leaves under normal conditions^6^.

As we have described for previous investigations, we identified meaningful biological processes and visualized significant metabolic pathways through GO enrichment and MapMan analyses^26, 63^. Results from the former suggested that biological processes mainly related to carbohydrate metabolism are important in the crosstalk between the drought up-regulation and diurnal rhythm. Other researchers have also indicated that carbohydrates are closely associated with water-deficit responses and the diurnal cycle. For example, levels of trehalose, a carbohydrate storage molecule, are elevated through manipulation of the intermediate trehalose-6-phosphate, which enables transgenic rice plants to become more drought-tolerant through sugar-signaling and carbohydrate metabolism^64^. However, only a few examinations have considered the strong association between drought stress and diurnal rhythm, as coupled with carbohydrate metabolism^10, 43, 65^. We also found that the GO term for embryo development had the highest fold-enrichment in genes that were up-regulated by both drought and diurnal rhythm. This connection might be explained by the processes of physiological desiccation and dormancy in rice that occur during embryo development prior to grain harvest^66^. In barley, the diurnal cycle has a critical role in the formation of caryopses^67^. The GO term with the second-highest fold-enrichment was related to the water-stress response.

Terms for light reactions and relative carbohydrate metabolism were also significantly represented in our analysis of genes that are both drought down-responsive and diurnally rhythmic. This is manifested in C3 plants by a reduction in CO_2_ diffusion through the stomata and mesophyll under drought conditions^68–70^. In *Phaseolus vulgaris*, the speed and the extent of photosynthetic recovery depends upon variations in the carbon balance^68, 71, 72^. Because 15% of relatively down-regulated genes in drought-tolerant *Thellungiella* are photosynthetic, it is highly probable that those genes also have key roles in light reactions^73^. However, future studies are required to determine whether these features are conserved in rice, and to learn how they might be connected to the drought mechanism via the diurnal cycle. In *Ricinus communis*, leaf development is influenced by the combination of drought stress and diurnal regulation^70^. Although those tissues grow more rapidly late at night than in the late afternoon, their diurnal amplitude in the rate of leaf growth decreases during periods of stress^74^.

Our MapMan results confirmed the findings from GO analysis that terms for the sucrose-starch pathway and light reactions are highly enriched. There, 10 genes allocated to the sucrose-starch pathway were up-regulated by drought while four genes related to photosynthesis were down-regulated (Fig. 5). Diurnal fluctuations in starch and sucrose contents under a water deficit are possibly influenced by the regulation of genes allocated to that pathway. Starch is accumulated during the daytime as a product of photosynthesis, but is then degraded at night to provide and export sugars to sink organs^43, 75^. Soluble sugars are accumulated in leaves when the water supply is limited in the growing environment. They can act as osmolytes to maintain normal transpiration and leaf-water content under drought conditions^42^. However, in periods of drought, starch is degraded to soluble sugars during daylight hours as well. Our findings here are supported by previous work with *Arabidopsis thaliana*, where the accumulation of sucrose at the end of the day is critical in protecting plants against xenobiotic and oxidative stresses^76^. We also speculated that the alteration of chlorophyll fluorescence under a water deficit is related to four genes allocated to photosynthesis (Fig. 6), possibly affecting PSII. Because photosynthesis is influenced by drought due to limitations in carbon uptake and CO_2_ diffusion, we might expect diverse metabolic changes to occur accordingly^47^. With regard to the diurnal cycle, photo-inhibition by strong light intensities is more like to happen mid-day, which then causes the photosynthesis potentiality in PSII to decline^45–47^. We might conclude that starch degradation in the daytime increases the level of soluble sugars, which then can compensate for drought-induced changes in photosynthate contents. Therefore, future experiments should also include the monitoring of changes in metabolites during daylight hours.

For our interaction network, we incorporated genes that are up-regulated by drought as well as those with diurnal patterns of expression. Several candidates showed the most potential for functional analysis, including *OsBAM3*, and *OsIsoamylase1* (*LOC_Os08g40930*). These were selected because one homolog from *Arabidopsis*, *AtBAM1* (*AT3G23920*), is down-regulated under drought stress and its expression is linked with diurnal degradation of starch to sustain proline biosynthesis during periods of osmotic stress. Furthermore, the *atbam1* mutant is rendered drought-tolerant because starch does not break down in its guard cells and its rate of stomatal opening is lower when compared with normal plants^77, 78^. Rice *granule-bound starch synthase1* (*LOC_Os06g04220*), which functions similarly to *OsIsoamylase1* in the pathway for starch biosynthesis, has decreased expression in the ABA-independent pathway under a water deficit^79^. Based on our findings here, we propose that starch synthesis is greater under drought conditions due to the upregulation of *ADP-glucose pyrophosphorylase large subunit 1* (*OsAGPL1*, *LOC_Os05g50380*), *starch synthase I* (*OsSSI*, *LOC_Os06g06560*), and *OsSBE3*. Consequently, higher levels of sucrose and soluble sugars might be turned over from enhanced starch contents through the expression of four candidate β-amylase genes (*OsBAM2*, *OsBAM3*, *OsBAM5*, and *LOC_Os03g22790*), plus an α-amylase precursor (*LOC_Os06g49970*), and *4-α-glucanotransferase* (*LOC_Os07g43390*) (Fig. 4). The expression of *OsBAM2, OsBAM3, and OsBAM5* were strongly induced in the end of night (Figures S4 and S5). In previous studies, expression of *AtBAM1* was the highest during the shifting period from dark to light^80^. However, the expression phase of *AtBAM1* is shifted to lateday in drought conditions^27^. Similarly, the expression of β-amylase genes in rice might have the shift of expression phase to daylight hours to produce increased amount of sucrose and soluble sugars in water deficient response. How the transcriptome of drought responsive genes in rice shifts according to diurnal pattern should be elucidated through further analyses.

OsbZIP23 could be responsible for regulating interactions between drought stress and diurnal rhythm. This TF contributes to drought tolerance, and it binds, targets, and regulates several genes encoding enzymes in the starch-sucrose pathway^57, 81^. In particular, drought-induced ABA inhibits OsPP2C49, and SAPK2 is then activated to phosphorylate OsbZIP23^57, 82^. Hence, the expression of drought-responsive genes is triggered by this activation of OsbZIP23^57^. Overexpression of OsbZIP23 in rice leads to a drought-tolerant phenotype and increased soluble sugar contents in the leaves of stressed plants^83^. The four β-amylases mentioned above are bound by OsbZIP23. Their function in breaking down starch might play a role in the drought response and could explain the elevated production of soluble sugars^84^. *OsAGPL1* is also bound by OsbZIP23, and activation of the latter causes the former to be up-regulated. Thus, transitory starch synthesis in the leaves might be driven by *OsAGPL1*. A drought-related decline in chlorophyll fluorescence might be eased by the overexpression of OsbZIP23 under water stress^81^. *Lhcb1* (*LOC_Os01g41710*) is bound by OsbZIP23 when water is limiting, and *LOC_Os05g28090*, which encodes chloroplast NAD(P)H dehydrogenase, is also regulated by OsbZIP23. Therefore, the downregulation of both of these genes, as controlled by OsbZIP23, might induce the photosynthetic and diurnal physiological differences in response to drought.

**Figure 8:**
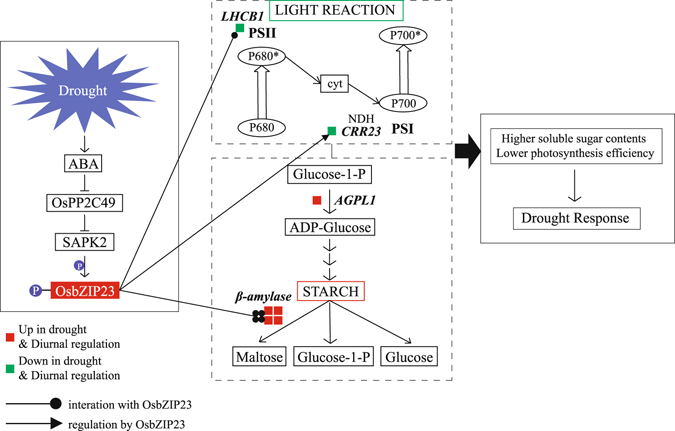
Suggested model to describe how drought-inducible OsbZIP23 regulates both light reactions and starch-sucrose pathway. Red boxes, genes showing diurnal expression pattern and up-regulated by drought; green boxes, diurnal genes down-regulated by drought. Genes encoding proteins interacting with OsbZIP23 are linked with circle-head lines; those encoding proteins regulated by bZIP23 are connected with triangle-head arrows. Pathways in solid-line rectangles were previously elucidated. Pathways bordered by rectangles with dotted lines include genes proposed here as part of crosstalk between diurnal expression and drought response.

In conclusion, we exploited the advantages of meta-expression analysis to discover a functional gene network that explains the cross-talk between diurnal rhythm and drought stress. Our results indicate that the starch-sucrose pathway and light reactions might be its main targets. Under a water deficit, the daytime response is critical to the growth of rice plants because higher starch turnover, even in daylight hours, is to sustain limited production of photoassimilates during drought periods. The OsbZIP23 TF, which is involved in the drought-response mechanism, binds to four *OsBAM*s that are up-regulated by drought and also expressed diurnally. This TF also binds to *OsAGPL1* in the starch-sucrose pathway and to the drought down-responsive and diurnal *OsLHCB1* in PSII. Physiological and metabolic changes to starch/sucrose production and photosynthesis potentiality are stimulated by OsbZIP23 in response to drought stress. Based on these findings, we have developed a model to illustrate this regulatory mechanism (Fig. 8). Nevertheless, more functional and molecular investigations are required if we are to obtain deeper insight into this type of crosstalk.

## Methods

### Analysis of drought-stress regulation and diurnal rhythms using publicly available microarray data

We downloaded nine raw data series containing 131 samples related to abiotic stress from the NCBI-GEO. This meta-expression database was then modified so we could focus on the effects of drought, cold, heat, and submergence. Using six dataseries (GSE6901, GSE21651, GSE24048, GSE25176, GSE26280, and E-MEXP-2401), we selected drought-responsive genes, then normalized the data using the Affy Package from R language and changed intensity values to a log2 scale^35, 85^ Average fold-change values, i.e., drought-stressed samples over control (MOCK) samples, were calculated. Genes that were up- or down-regulated by diverse drought conditions were sorted by KMC via MeV software. The average values computed for selected genes are imaged in the heatmap in Fig. 1 (left panel). We also conducted meta-expression analysis of genes that were either up-regulated (712) or down-regulated (761) according to a diurnal pattern. Using RXP_0002 data (GSE36040, GEO), we analyzed expression patterns of those genes in leaf blades sampled at 2-h intervals for 2 d during nine developmental stages for field-grown plants^22^. After the data were log2-normalized, KMC analysis was performed to delineate the genes that were differentially expressed diurnally. This produced seven gene groups, for which we produced heatmap images (MeV software) and then edited with Adobe Photoshop and Illustrator CS6^36^. Moreover, we divided the genes to four groups according to their differential rhythmic patterns. The average of normalized values from the diurnal data of four time phases (Midday, 08:00–16:00; Lateday, 18:00–20:00; Midnight, 22:00–02:00; and Predawn, 04:00–06:00) were calculated to identify the peak of expression level.

### Analysis of GUS expression

Screening of the promoter trap lines and GUS staining were performed as described previously^37, 38, 86^. We used a T-DNA-specific primer in the pGA2715 vector as well as gene-specific primer sequences to confirm, via PCR, the co-segregation of GUS expression and T-DNA insertion in rice Line PFG_3A-02918 (Table S8). The promoter for the GUS vector was constructed using the pGA3519 binary vector, as described previously^87^. For observing drought-inducible GUS activity in transgenic rice, we stained leaves from MOCK control and drought-stressed plants at the same time interval. Expression of GUS in the shoots was examined with an SZX16 microscope (Olympus, Japan). As negative controls for promoter trap systems of drought inducible expression patterns, we used T-DNA insertion in rice Line PFG_4A-04197 and PFG_5A-00191 to confirm the GUS activities controlled by the promoter trap of drought unresponsive genes. PFG 4A-04197 is a promoter trap line of *LOC_Os03g08010* and PFG 5A-00191 is a promoter trap line of *LOC_Os03g01910*. Both genes showed ubiquitous expression and are not in our candidate gene list (Figure S3)^38^.

### Plant materials and growing conditions

For RNA extractions and GUS assays, we germinated seeds of *Oryza sativa* L. ssp. *japonica* cv. Dongjin on Murashige and Skoog (MS) media and grew the seedlings for 10 d^50^. Conditions in the climate-controlled chamber included 28 °C/22 °C (day/night), a 12-h photoperiod, and light provided by incandescent lamps so we could monitor diurnal gene expression. Samples were collected at the end of the day (ED; 19:30), the middle of the night (MN; 1:30), the end of the night (EN; 7:30), and the middle of the day (MD; 13:30). Plants grown for 10 d under continuous light (28 °C) were then subjected to drought stress by withholding water for 3, 6, or 12 h while those assigned to the MOCK control continued to receive normal irrigation.

For the starch-sucrose assay, plants were grown for five weeks in a controlled-climate chamber from seeds germinated in MS media. After 9 d of exposure to drought conditions, samples were collected at the ED and EN time points. Two-month-old seedlings grown in a greenhouse were used for evaluating chlorophyll florescence. Afterward, the plants were moved to the climate-controlled chamber for acclimation before the stress treatments began. After 4 d of exposure to drought conditions, samples were collected at the MN and MD time points. At least three biological replicates were prepared and performed for each analysis.

### Quantitative RT-PCR analysis

We extracted total RNA from whole seedlings and synthesized cDNA using methods introduced previously^88, 89^. The quality of cDNA samples was validated and evaluated with two drought-responsive marker genes (*Oryza sativa dehydration-responsive element binding transcription factor 2b*, or *OsDREB2b*, and *OsbZIP23*), the daytime-expressed *Oryza sativa Late Elongated Hypocotyl* (*OsLHY*), and the nighttime-expressed *Heading Date 1* (*HD1*) (Figure S6)^90–93^. Quantitative real-time PCR analysis was conducted with Prime Q-Master Mix (2X, Real-time PCR with SYBR Green I) (GeNet Bio, Korea) and the Rotor-Gene 6000 (Qiagen, Hilden, Germany). The rice *UBIQUITIN 5* (*OsUbi5*, *LOC_Os01g22490*) and rice *UBIQUITIN 1* (*OsUbi1*, *LOC_Os03g13170*) served as a housekeeping gene for normalization, based on the comparative Ct (2^−∆∆Ct^) method^50, 94, 95^. All internal controls and gene-specific primer sequences are shown in Table S8. The P-values were calculated along a two-tailed distribution and two-sample unequal variance.

### Analysis of gene ontology enrichment

The biological roles of selected genes were investigated through GO enrichment analysis of information from ROAD^85^. By using the GO enrichment tool installed in ROAD, GO terms were mapped on to the query genes with the detailed information. We generated the fold enrichment value by calculating the ratio of the query number value to the query expected value. Enriched GO terms that met our criteria of ≥two-fold enrichment and P ≤ 0.05 are shown in Fig. 3 and in Tables S4 and S5. Hyper P-values based on a hypergeometric distribution were provided by the GO enrichment tool installed in ROAD.

### MapMan analysis

Candidate genes were analyzed through the Metabolism, Sucrose-Starch, Plant Glycolysis, and Photosynthesis overviews contained in the MapMan toolkit^40^ (Fig. 2). Additional analyses for regulation, transcription, large enzymes, cellular-responsive, secondary metabolism, receptor-like kinases, and proteasomes were also covered (Figures S7 and S8).

### Measurements of starch and soluble sugar contents

We measured starch and soluble sugar contents by an enzymatic method^41, 65^. After fresh weights (FWs) were determined from each sampled leaf, they were ground with liquid nitrogen in a TissueLyser II (Qiagen, Germany). Extractions were performed with 10% perchloric acid. Following neutralization and centrifugation (13,000 rpm, 15 min), the aqueous phase was used for measuring soluble sugars while the sediment was used for starch. All values were calculated to the nearest mg g^−1^ FW of sample, based on the tissue weight and solution volume that was used with the enzymatic method.

### Measurement of chlorophyll fluorescence

Chlorophyll fluorescence was evaluated using Handy PEA (Hansatech Instruments, England). The leaf samples were first dark-adapted for 15 min by closing the shutter plates of the leaf clips. Values were measured at a maximum light intensity of 3000 μm olm^−2^ s^−1^, and P-values were calculated based on a two-tailed distribution and two-sample unequal variance.

### Analysis of interaction network

We proposed a functional gene network of 276 genes that encode enzymes for whole starch synthesis and the degradation pathway, using information from the RiceCyc and the Rice Interaction Viewers. Genes were ordered according to steps in each pathway. Any genes in the pathways that showed diurnal rhythm and drought-responsiveness but which were not part of these interaction results were added to the network as nodes (see representations in Fig. 7). We then added genes that were bound, targeted, and/or regulated under drought stress by OsbZIP23 TF (GEO; GSE81462)^57^. The network was edited with Cytoscape program (http://www.cytoscape.org/).

### Data Availability

All data analyzed during this study are included in this published article and its supplementary information files.

## Electronic supplementary material

Supplementary Information

Dataset 1

Dataset 2

Dataset 3

Dataset 4

Dataset 5

Dataset 6

Dataset 7
