# Supplementary material for: Crosstalk between diurnal rhythm and water stress reveals an altered primary carbon flux into soluble sugars in drought-treated rice leaves
Source: Sci Rep. 2017 Aug 15;7:8214. doi: 10.1038/s41598-017-08473-1 (PMC5557844; doi:10.1038/s41598-017-08473-1)
Supplement: Supplementary file 1 — Supplementary Information [file 41598_2017_8473_MOESM1_ESM.pdf]

Crosstalk between diurnal rhythm and water stress reveals an altered primary carbon flux into soluble sugars in drought-treated rice leaves

Seo-Woo Kim[1,2], Sang-Kyu Lee[1,2], Hee-Jeong Jeong[1] , Gynheung An[1] ,Jong-Seong Jeon[1,*]and Ki-Hong Jung[1,*]

[1] Graduate School of Biotechnology & Crop Biotech Institute, Kyung Hee University, Yongin 446-701, Korea
[2] These authors contriuted equally to this work
* Corresponding author: Jung, Ki-hong (khjung2010@khu.ac.kr) and Jeon Jong-Seong (jjeon@khu.ac.kr), Fax: 82-31-201-3178, Tel: 82-31-201-3474

Supplementary Figures

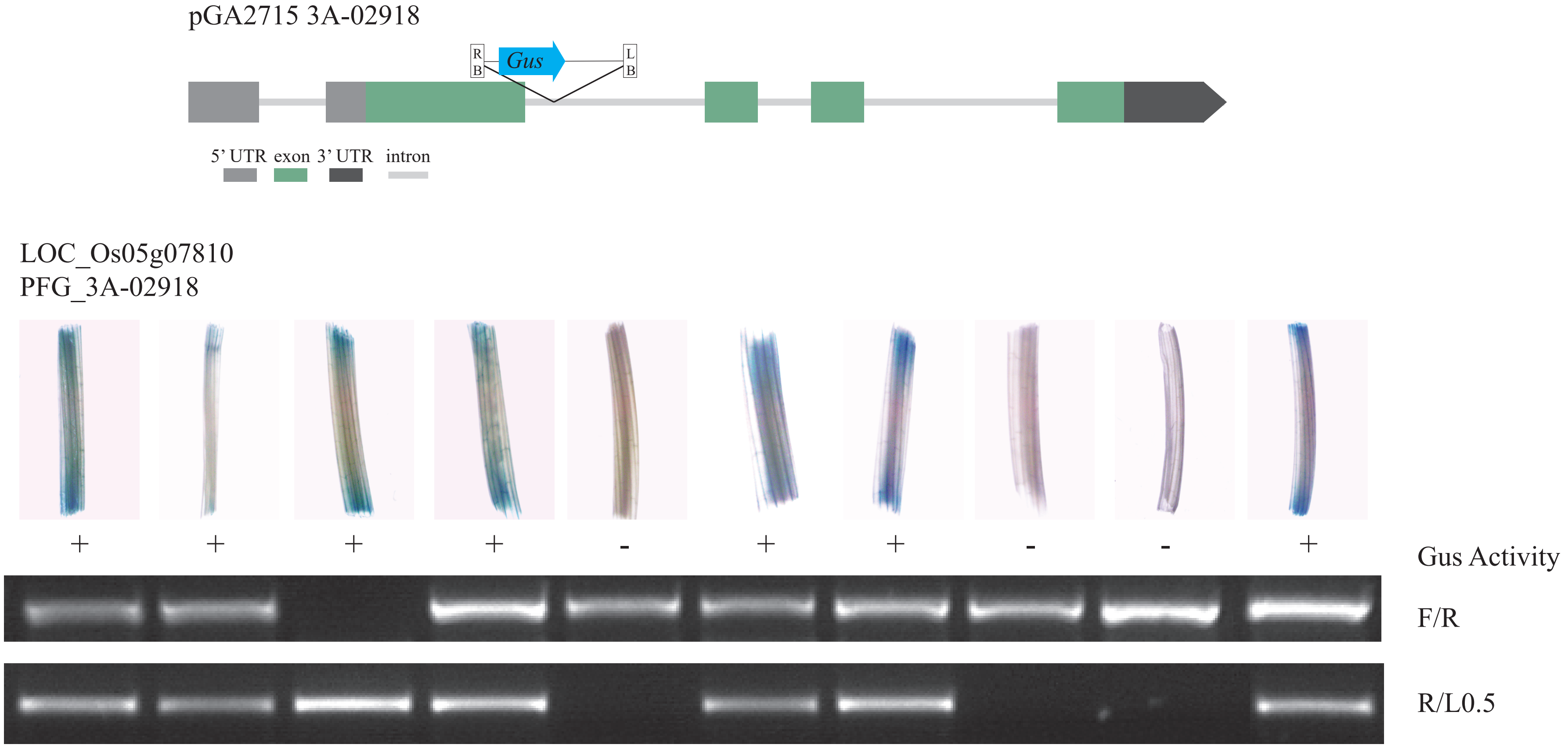

Supplementary figure S1. Information about T-DNA Line 3A-02918 in pGA2715 vector used for promoter gene trap system. Confirmation of gus expression patterns was matched to its co-segregated genotype.

a promoter trap line of LOC_Os05g07810 (two more replicates)

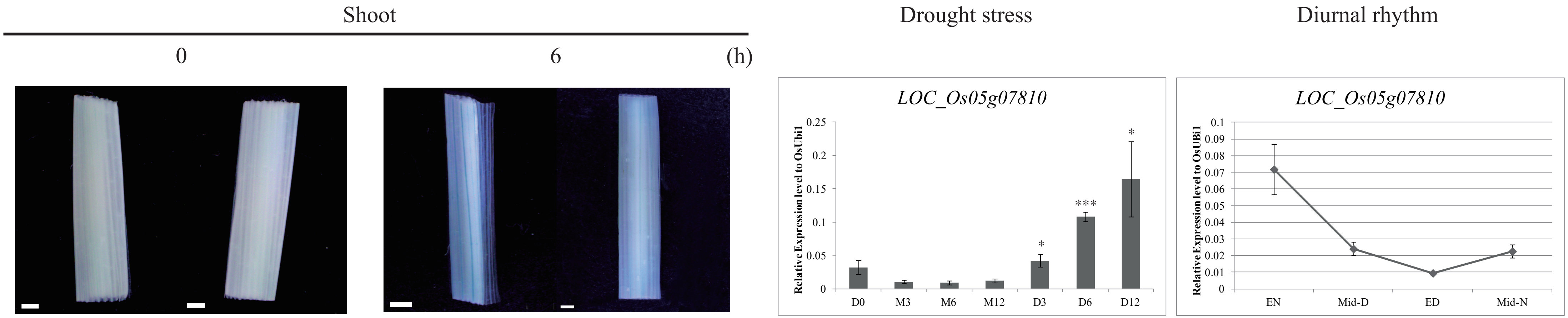

b promoter-GUS line of LOC_Os10g42610 (two more replicates)

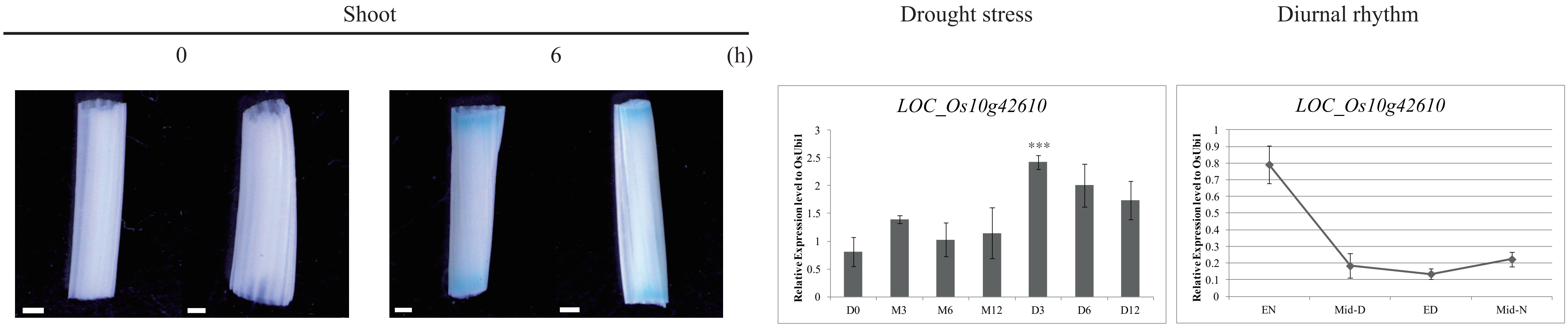

Supplementary figure S2. Biological replicates of drougnt-inducible GUS expression and expression level under drought stress and diurnal relative to *OsUbi1* as another housekeeping gene for *LOC_Os05g07810* (a) and *LOC_Os10g42610* (b) from Figure 2.

a promoter trap line of LOC_Os03g08010

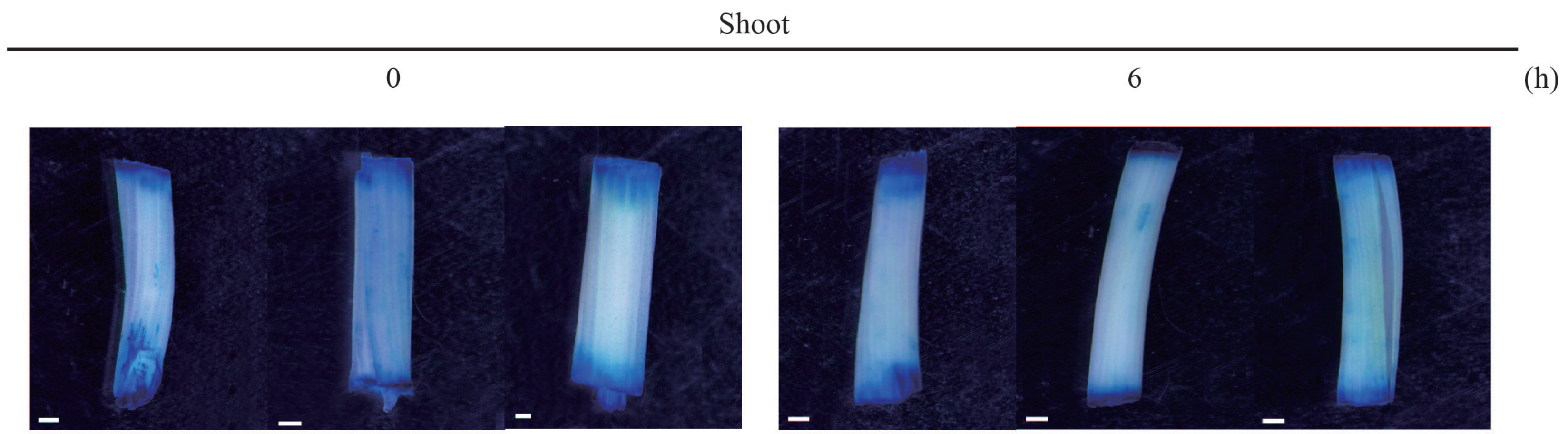

b promoter trap line of LOC_Os03g01910

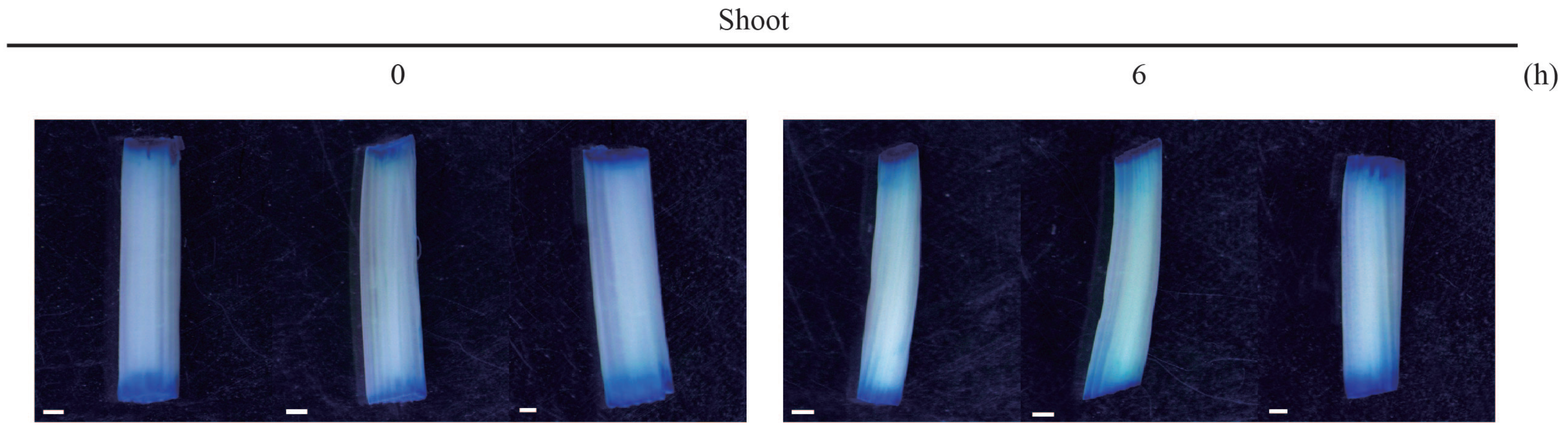

Supplementary figure S3. Expression patterns of GUS through the promoter trap system with ubiquitously expressed genes *LOC_Os03g08010* (a) and *LOC_Os0 3g01910* (b) in drought-treated conditions.

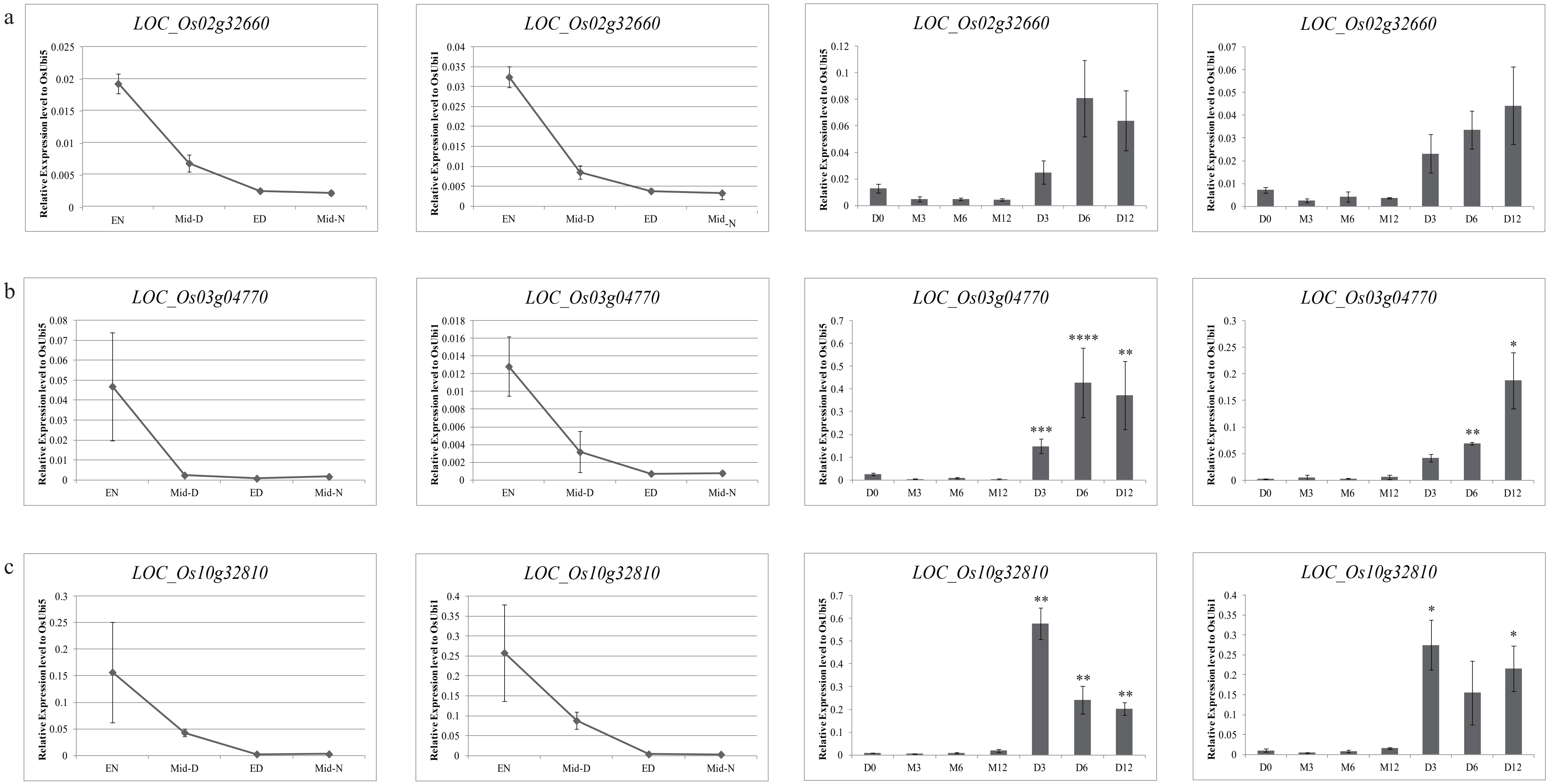

Supplementary figure S4. Expression patterns under drought stress and diurnal rhythm relative to *OsUbi5* and *OsUbi1* for our candidates *LOC_Os02g32660* (a), *LOC_Os03g04770* (b), and *LOC_Os10g32810* (c).

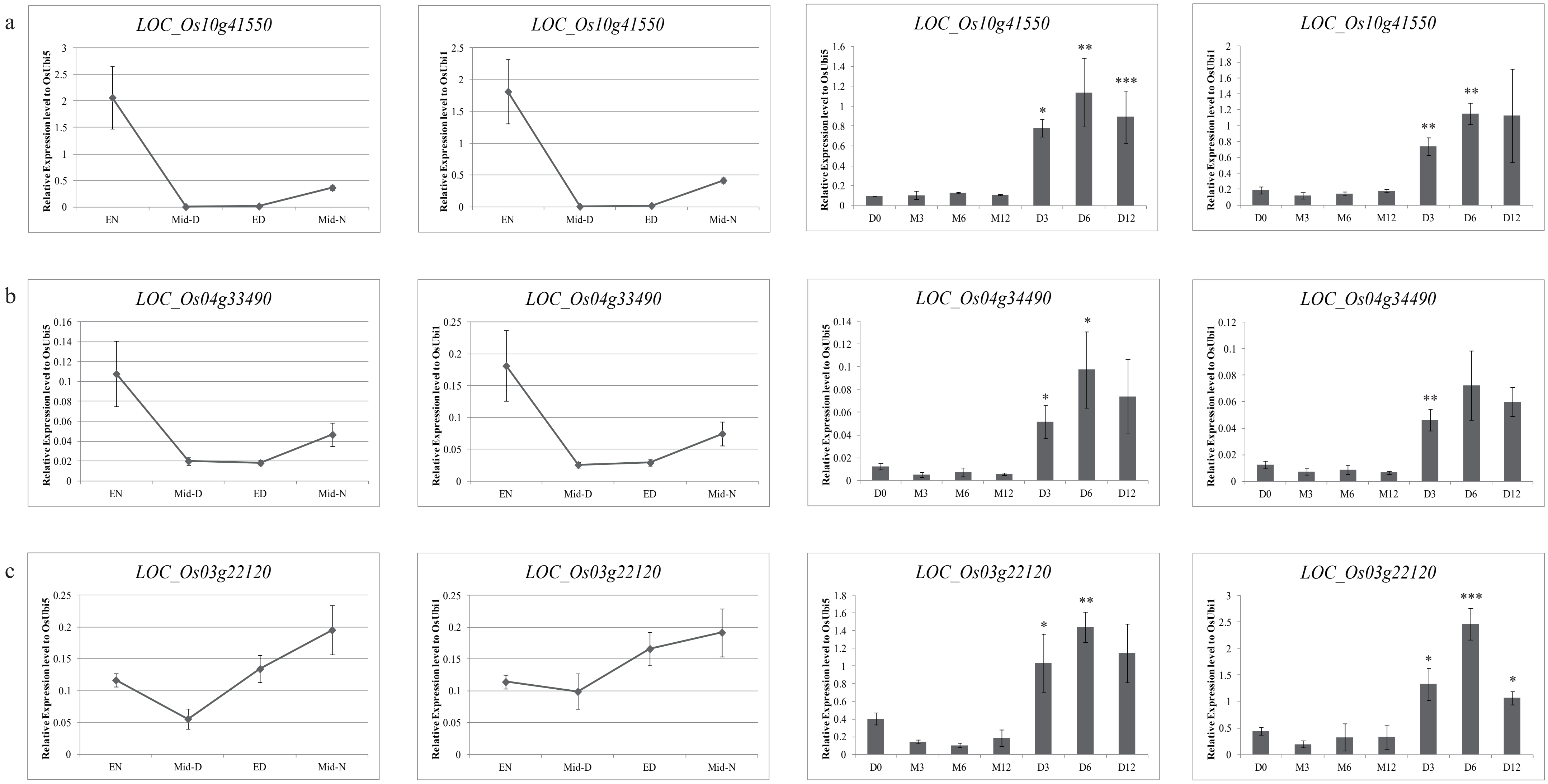

Supplementary figure S5. Expression patterns under drought stress and diurnal rhythm relative to *OsUbi5* and *OsUbi1* for our candidates *LOC_Os10g41550* (a), *LOC_Os04g33490* (b), and *LOC_Os03g22120* (c).

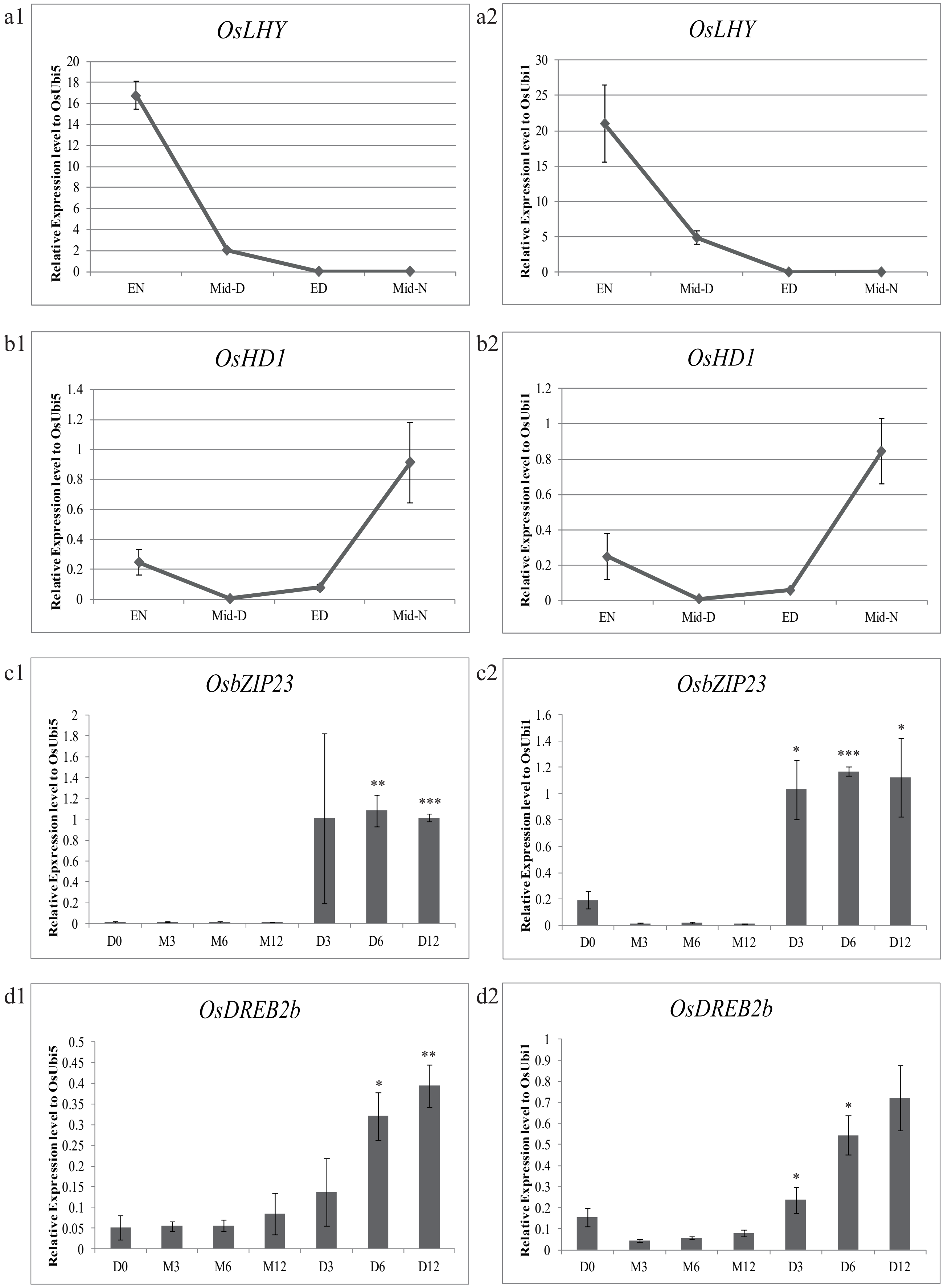

Supplementary figure S6. Validation of cDNA sample quality using qRT-PCR with diurnally expressed *OsLHY* (a) and *HD1* (b), as well as with *OsbZIP23* (c) and *OsDREB2b* (d), both of which are drought-induced marker genes.

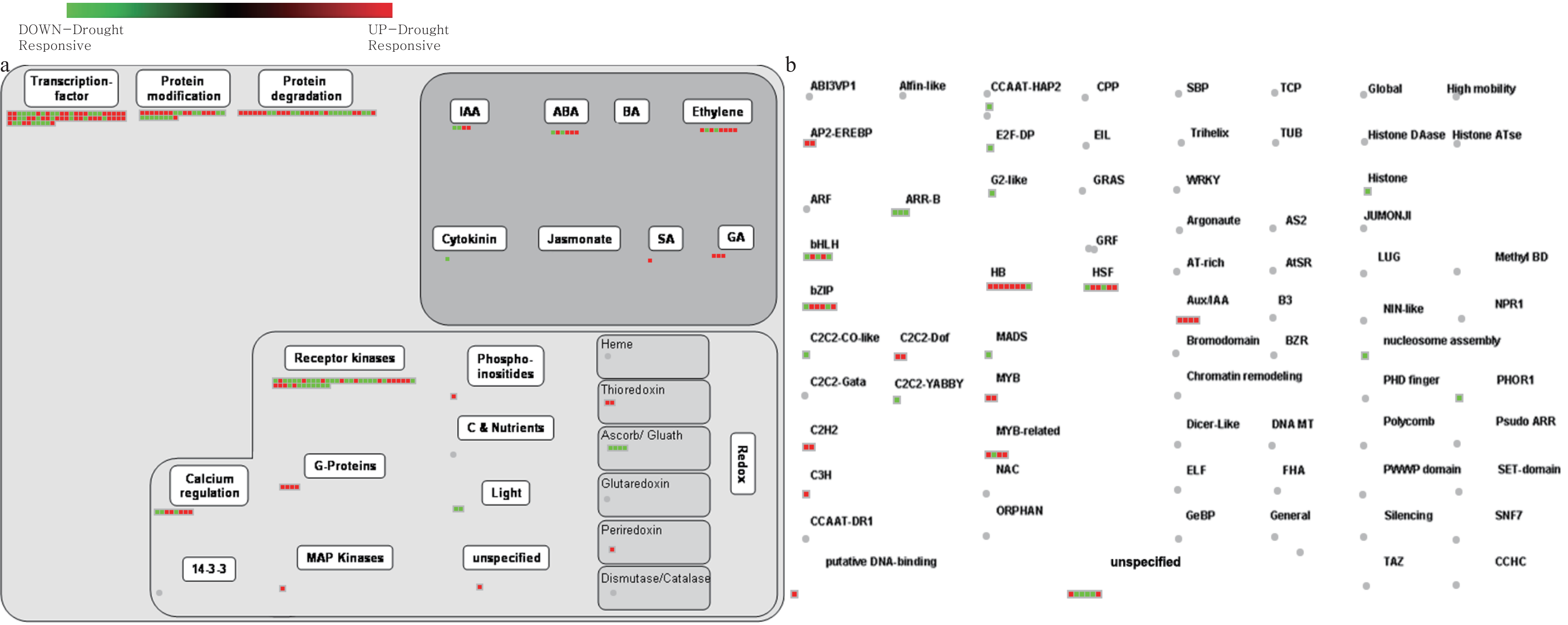

Supplementary figure S7. MapMan analysis of 766 drought-responsive genes that also show diuranl rhythm. In Regulation overview (a), and Transcription overview (b), genes marked with red or green are up-regulated or down-regulated, respectively, by drought.

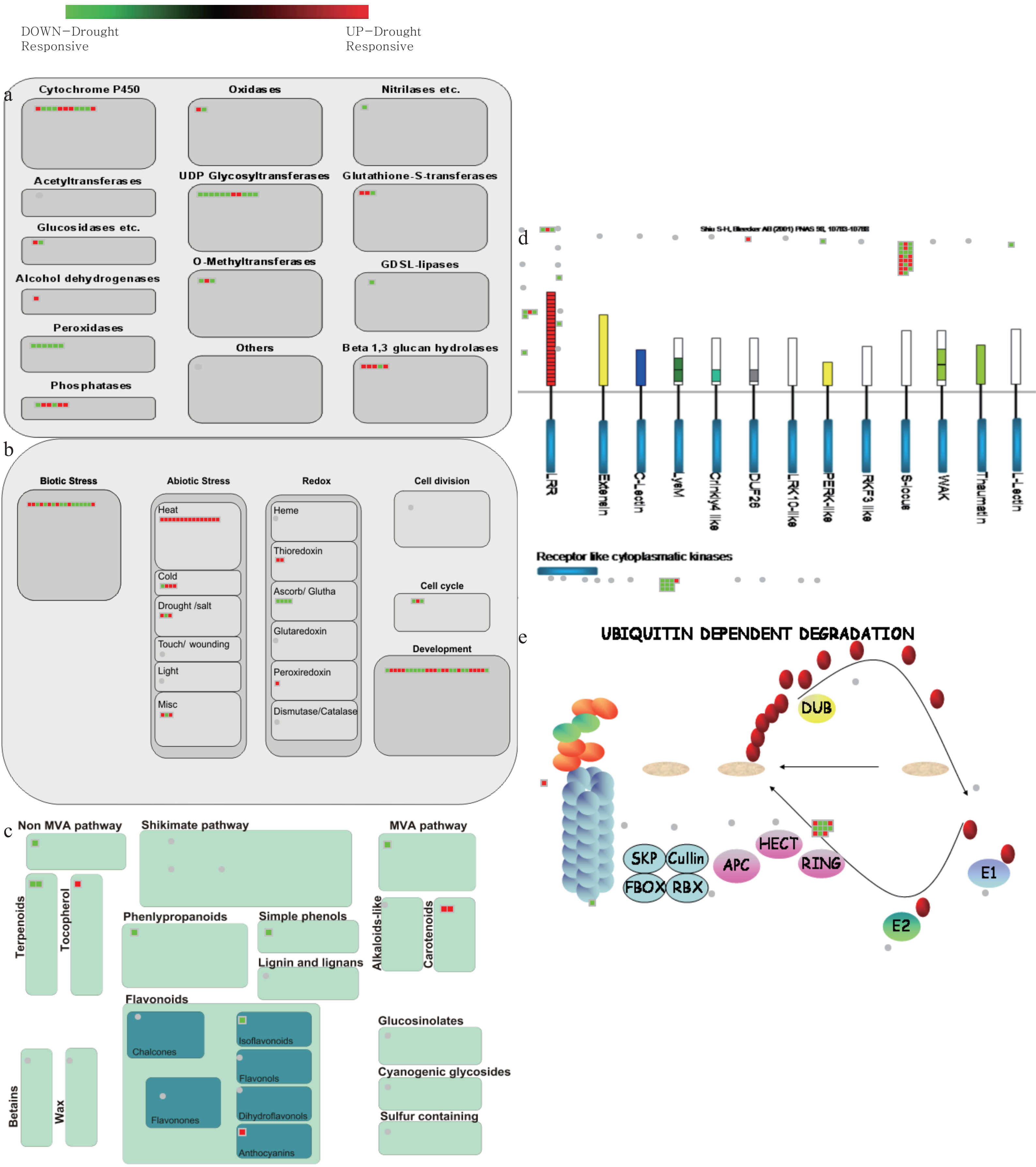

Supplementary figure S8. MapMan analysis of 766 drought-responsive genes that also show diuranl rhythm. In Large Enzyme overview (a), Cellular-Responsive overview (b), Secondary Metabolism overview (c), Receptor-like Kinases overview (d), and Proteasome overview (e), genes marked with red or green are up-regulated or down-regulated, respectively, by drought.

Supplementary Tables

The data of supplementary tables S1, S2, S3, S4, S5, S6, and S7 are provided in separate excel files

Supplementary table-S8. Primer Sequences used for genotyping and quantitative RT-PCR analyses

Purpose	MSU_locus_IDs	vector	primer direction	Primer sequence	Gene
genotyping	LOC_Os05g07810	PFG_3A-02918	Forward	GCAACGACACCTGCAGGCTA	universal stress protein domain containing protein
genotyping	LOC_Os05g07810	PFG_3A-02918	Reverse	AGACGATCGGAATCCTCACC	universal stress protein domain containing protein
genotyping	LOC_Os05g07810	pGA2715_L0.5	Forward	TTGGGGATCCTCTAGAGTCGAG	universal stress protein domain containing protein

Purpose	MSU_locus_IDs	RAP_locus_IDs	primer direction	Primer sequence	Gene
qRT-PCR	LOC_Os05g07810	Os05g0170200	Forward	GCAACGACACCTGCAGGCTA	universal stress protein domain containing protein
qRT-PCR	LOC_Os05g07810	Os05g0170200	Reverse	TGACGTGGACAAGCACCAGC	universal stress protein domain containing protein
qRT-PCR	LOC_Os01g22490	Os01g0328400	Forward	GCACAAGCACAAGAAGGTGA	UBI5
qRT-PCR	LOC_Os01g22490	Os01g0328400	Reverse	GCCTGCTGGTTGTAGACGTA	UBI5
qRT-PCR	LOC_Os03g13170	Os03g0234200	Forward	CACCCTGGCTGACTACAACA	UBI1
qRT-PCR	LOC_Os03g13170	Os03g0234200	Reverse	ACACTTCTTCTTGCGGCAGT	UBI1
qRT-PCR	LOC_Os10g42160	Os10g0571900	Forward	AGGCAGATCGAGACACGATC	expressed protein
qRT-PCR	LOC_Os10g42160	Os10g0571900	Reverse	CGTCCGTCCGCACTTGAACT	expressed protein
qRT-PCR	LOC_Os08g06110	Os08g0157600	Forward	GAAGCGGTGGTAAAGGTGAG	LHY
qRT-PCR	LOC_Os08g06110	Os08g0157600	Reverse	CCTGGAGAAGTGCCATTGTT	LHY
qRT-PCR	LOC_Os06g16370	Os06g0275000	Forward	CTACTCAGGGTGCCCATCAT	HD1
qRT-PCR	LOC_Os06g16370	Os06g0275000	Reverse	CAGCAGTGCCAAGATCACTC	HD1
qRT-PCR	LOC_Os02g52780	Os02g0766700	Forward	ATCGAGAAGGTTGTCGAGAG	bZIP23
qRT-PCR	LOC_Os02g52780	Os02g0766700	Reverse	ATCGTTCAGCTCCTTAAGTT	bZIP23
qRT-PCR	LOC_Os05g27930	Os05g0346200	Forward	TATCAGCATGCGAGTCCACT	DREB2B
qRT-PCR	LOC_Os05g27930	Os05g0346200	Reverse	TCATCATAGGTAACCGATTC	DREB2B
qRT-PCR	LOC_Os02g32660	Os02g0528200	Forward	ATGCCGTCGAAGATTTGACC	glycogen branching enzyme

qRT-PCR	LOC_Os02g32660	Os02g0528200	Reverse	AACAACCAACACGGTAGTCA	glycogen branching enzyme
qRT-PCR	LOC_Os03g04770	Os03g0141200	Forward	ACGTGCGTGGAGATGCGCAA	β-amylase3
qRT-PCR	LOC_Os03g04770	Os03g0141200	Reverse	ACCTGCTCGCGGCACACGTC	β-amylase3
qRT-PCR	LOC_Os10g32810	Os10g0465700	Forward	GGAGGAAAACCGCTCTGGAG	β-amylase2
qRT-PCR	LOC_Os10g32810	Os10g0465700	Reverse	ATTCCCCAAGCGTGACAACA	β-amylase2
qRT-PCR	LOC_Os10g41550	Os10g0565200	Forward	CCCATCCAGGTCTACTCCGA	β-amylase5
qRT-PCR	LOC_Os10g41550	Os10g0565200	Reverse	TGGCCTCAGGATACGAAGGA	β-amylase5
qRT-PCR	LOC_Os04g33490	Os04g0409900	Forward	TTGTTCCACTTGACGGGGAC	neutral/alkaline invertase
qRT-PCR	LOC_Os04g33490	Os04g0409900	Reverse	GAACCGACAGATCGCCTGAA	neutral/alkaline invertase
qRT-PCR	LOC_Os03g22120	Os03g0340500	Forward	AAAACGCTAGGCTGAGGGAG	sucrose synthase
qRT-PCR	LOC_Os03g22120	Os03g0340500	Reverse	ATCCAACGGAACTGCCCAAA	sucrose synthase
